# A novel multimodal AI framework for early diagnosis of idiopathic Parkinson’s disease

**DOI:** 10.1007/s11517-026-03547-7

**Published:** 2026-03-13

**Authors:** Efe Yılmaz Taşyürek, Şaban Murat Altun, Ata Emir Uncu, Sefa Tunca, Sevinç İlhan Omurca, Meltem Kurt Pehlivanoğlu, Aybala Neslihan Alagöz, Oğulcan Kalkan

**Affiliations:** 1 Department of Computer Engineering, Kocaeli University, Kocaeli, Turkey; 2 Department of Neurology, Kocaeli University Training and Research Hospital, Kocaeli, Turkey

**Keywords:** Parkinson’s disease, Early diagnosis, Multimodal classification, Decision support system, Artificial intelligence, Neurodegenerative disorder detection

## Abstract

**Abstract:**

Parkinson’s disease (PD) is a progressive neurodegenerative disorder marked by motor symptoms, but early diagnosis is challenging due to symptom overlap with other conditions and a lack of definitive biomarkers (clinical assessments). In this study, we propose a novel multimodal artificial intelligence (AI)-based decision support system aimed at the early diagnosis of idiopathic PD. To the best of our knowledge, this is the first framework to enable the synchronous analysis of four distinct modalities: walking, facial expression, voice, and posture, whereas prior studies have typically focused on unimodal or partially multimodal approaches. We also constructed a new dataset by establishing a controlled clinical testing environment equipped with an L-shaped walking track and an integrated audiovisual recording system to capture natural walking, turning, facial, vocal, and postural characteristics. For each modality, specialized AI models were developed and evaluated. For the walking modality, the proposed Bidirectional GRU model achieved the best performance in terms of both $F_1$ score (92.74%) and area under the curve (AUC) (97.86%), demonstrating superior gait-based classification performance. Similarly, in the face modality, the ensemble model integrating eXtreme Gradient Boosting (XGBoost), Random Forest (RF), and Categorical Boosting (CatBoost) yielded the highest $F_1$ score (92.31%) while also achieving the best AUC (97.96%). For the voice and posture modalities, although the highest $F_1$ scores were not obtained, the RF-based models achieved the highest AUC values (99.85% and 97.56%, respectively) within their respective modality comparisons in the literature, reflecting strong class separability and discriminative capability.

**Graphical abstract:**

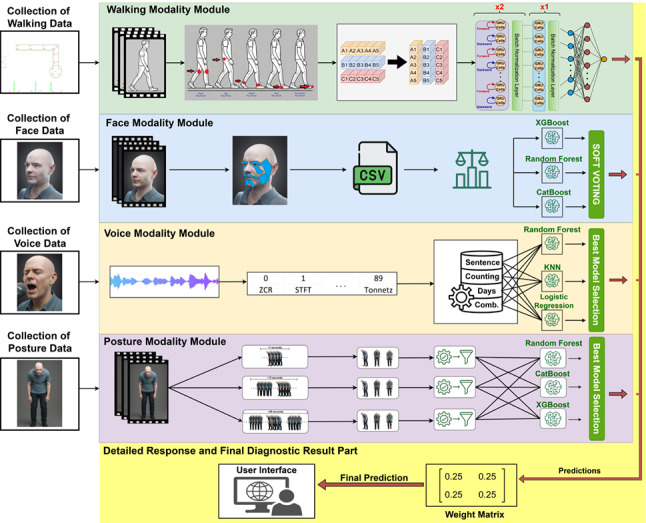

## Introduction

PD is among the most common neurodegenerative disorders, primarily affecting older adults, and its prevalence is expected to continue increasing. It is a neurological disorder characterized by impaired motor control, resulting from the progressive loss of brain cells over time. The disease is clinically characterized by four cardinal motor symptoms: bradykinesia (slowness of movement), tremor, muscular rigidity, and postural instability (balance impairment). In addition to these, one of the hallmark features of Parkinsonism includes motor freezing (also known as motor blocks), a form of akinesia that results in temporary inability to initiate or continue movement. The diagnosis of PD is typically based on neurological examination, assessment of the patient’s symptoms, and certain laboratory tests. However, these conventional methods present various challenges and limitations. Accurate diagnosis can be challenging in the early stages of the disease. Currently, the diagnosis of PD relies primarily on clinical findings, as there is no definitive diagnostic test available. As a result, existing diagnostic approaches often require specialist expertise and can be time-consuming. Therefore, there is a critical need for new methods that enable faster and earlier diagnosis. In particular, diagnosing PD during its prodromal or early stages remains a significant challenge.

Machine learning (ML) models have tremendous potential to transform medical diagnostics by enabling earlier detection, improving precision, and supporting clinicians [1]. In this context, AI- and ML-assisted early detection of PD has emerged as a promising area of research. However, while AI and ML-driven approaches can achieve high Parkinson’s disease diagnostic accuracy, several challenges remain in translating these models into clinical practice. These include the need for real patient validation, model explainability, and frameworks that align with clinical workflows, highlighting the broader gap between data-driven ML performance and clinically grounded decision support [13].

In response to these challenges, this study proposes a multimodal AI-based decision support system that integrates multiple clinical assessments to enhance the diagnostic process for PD. The core novelty of the proposed approach lies in the synchronized and parallel analysis of four distinct modalities—walking, facial expression, voice, and posture—designed to maximize their complementary diagnostic value. To the best of our knowledge, this is the first study to concurrently evaluate all four modalities within a single, unified framework for PD detection. The selection of these modalities was guided by expert consultation and preliminary clinical evaluations conducted in close collaboration with an experienced neurologist. Specifically, walking abnormalities, facial masking, vocal tremor, and postural instability were identified as clinically meaningful markers with high diagnostic relevance. Each modality was selected based on both its established presence in the literature and its routine use in clinical neurological assessments. All data collection, annotation, and modeling procedures were conducted in collaboration with the Department of Neurology at Kocaeli University, ensuring strong clinical validity and real-world applicability. For each modality, a dedicated signal processing and ML pipeline was developed. Moreover, experimental results demonstrate that the proposed framework achieves the highest AUC values across all four modalities compared with existing studies in the literature, indicating superior class separability. In particular, for the walking and face modalities, the proposed models also achieved the highest $F_1$ scores, together with the best AUC values, reflecting both strong discriminative capability and balanced classification performance. The final diagnostic decision is obtained by integrating the outputs of modality-specific classifiers using a scoring-function-based fusion strategy, allowing each modality to contribute independently to the overall assessment. Unlike previous studies that predominantly analyze unimodal data or limited modality combinations, the proposed framework offers a comprehensive, clinically grounded, and quantitatively validated approach for the digital diagnosis of PD, supporting robust and reliable early-stage screening in clinical practice.

### Motivation and contributions

In the early stages of PD, accurate diagnosis is particularly challenging due to the overlap of its symptoms with those of other neurological disorders. Current clinical diagnostic methods rely heavily on expert evaluation, making the process both time-consuming and susceptible to interpretative variability. The dependence of diagnoses on subjective assessments increases the risk of incorrect decisions, which can lead to serious financial and emotional consequences for patients. These challenges highlight the need for the development of faster, more objective, reliable, and clinically interpretable systems.

The primary motivation of our study is to address this need and to develop a comprehensive decision support system that integrates and analyzes multiple clinical assessments of PD, namely walking, facial expression, voice, and posture. There is no existing study in the literature that simultaneously addresses all four of these core modalities for Parkinson’s diagnosis. Our work fills this critical research gap and enhance both diagnostic accuracy and clinical reliability in early detection. To achieve this, we established a controlled test environment featuring an L-shaped walking track to evaluate natural walking and turning ability, along with an integrated audio and visual recording infrastructure. The entire data collection process was conducted in collaboration with clinical experts to ensure the medical validity and reliability of the system. To the best of our knowledge, this study introduces the first dataset that concurrently captures and integrates these four key modalities for PD assessment. Moreover, another key contribution of this study is its advancement toward the integration of artificial intelligence based decision support systems into clinical diagnostic workflows. By jointly evaluating visual, auditory, and kinematic data, the system establishes a robust foundation for the early and accurate diagnosis of PD. Beyond its immediate application, the proposed framework is designed to serve as a foundation for broader use in the digital diagnosis of various neurological disorders.

### Organization

Section 2 provides a comprehensive review of the literature on PD based on symptom modalities used in the AI models for PD detection. In Section 3, we describe the data collection process involving individuals with PD and healthy participants, along with the creation of the new dataset. Section 4 provides a comprehensive description of the proposed modules within the PD detection framework, followed by the experimental results. Section 5 presents details of the system integration of the framework. Finally, the paper is concluded in Section 6.

## Related works

During the evaluation of existing literature on PD detection, the studies were systematically categorized into groups, each of which is discussed in detail below.

### Gait (walking)-based studies

Gait (or walking) analysis is one of the most extensively explored domains in the detection of PD using AI models. Tian et al. [12] developed a Cross-Spatiotemporal Graph Convolutional Network (CST-GCN) to model Parkinsonian gait from a dataset of 2,314 skeleton sequences collected from 102 PD patients and 46 healthy individuals using the Azure Kinect system. Although the model achieved a moderate accuracy of 67.7% by capturing spatial and temporal gait patterns, it relied exclusively on skeleton-based data, omitting visual, auditory, and postural modalities. The hospital setting also limits the ecological validity of the findings. Zeng et al. [15] improved upon traditional gait analysis by proposing a skeleton-silhouette fusion model trained on smartphone-recorded videos from 54 early-stage PD patients and 26 healthy controls. Their model achieved an accuracy of 71.25% by combining feature vectors from both silhouette and skeleton streams, but similarly neglected other symptom dimensions such as voice or facial expression. Zhao et al. [17] used MediaPipe to extract joint coordinates from gait videos recorded via smartphones and analyzed these using a ResNet50 architecture. Their model reached 92.86% accuracy for healthy individuals and 73.33% for PD patients, focusing specifically on leg joint data. However, it did not incorporate the temporal dynamics of movements or any additional symptom modalities. Di Biase et al. [2], adopting a sensor-based approach, analyzed kinematic and dynamic gait features from four patient cohorts (108 PD, 88 healthy) using pressure-sensitive insoles. The study found kinematic features more sensitive than dynamic ones, yet the high cost and limited accessibility of the hardware may hinder broader clinical use. Additionally, the analysis excluded other PD symptoms such as speech or facial deficits. Finally, Mehta et al. [9] introduced a label-free video analysis framework to predict UPDRS sub-scores for bradykinesia (BRADY) and postural instability/gait disorder (PIGD) from sit-to-stand transition videos. Trained on data from 32 PD patients, the model achieved F1-scores of 75% for BRADY and 78% for PIGD. However, its focus on a single type of motor transition limited the scope of motor symptom representation, excluding complex gait or turning movements.

### Facial expression-based studies

Facial expression analysis has emerged as a viable approach to detect non-verbal symptoms of PD, particularly hypomimia and masked facial expression. Xu et al. [14] specifically targeted hypomimia—reduced facial expressiveness common in PD—by applying deep learning algorithms to a dataset of 106 facial videos. The model achieved an accuracy of 81.73%, indicating good performance in symptom-specific detection. However, the study was symptom-narrow and did not examine motor or speech-related manifestations of the disease. Huang et al. [7] focused on masked face syndrome using a dataset of 95 PD patients. Their model employed StarGAN to generate synthetic facial expressions for data augmentation, achieving accuracy rates of 93.71% without synthesis, 97.04% without screening, and 99.53% when both techniques were combined. Notably, the best-performing model in their study was EfficientNet-B7, which achieved 100% accuracy for detection and 70.08% accuracy for facial expression recognition. Despite the high performance, the use of synthetic data raises concerns regarding clinical validity, as it may fail to capture the true heterogeneity of PD-related facial symptoms. Additionally, the study was limited to facial analysis and did not incorporate other symptom modalities. Lim et al. [8] is the only study that incorporates both facial expressions and voice characteristics. The researchers collected data via smartphones to extract visual and acoustic clinical assessments, applying nine different ML algorithms. They achieved an AUROC of 90% in distinguishing PD patients from the control group. However, when relying solely on facial data, the best-performing model—RF—achieved an AUROC of 69%. Despite the advantages of multimodal analysis, the study did not consider key motor symptoms such as gait and postural instability, which are essential for a comprehensive assessment of PD.

### Voice-based studies

Voice impairments in PD, such as dysarthria and reduced prosody, have been explored using various ML approaches. Di Cesare et al. [4] developed a speech analysis model using voice recordings from the MDVR-KCL dataset. Features such as Mel-Frequency Cepstral Coefficients (MFCC) and Gammatone Cepstral Coefficients (GTCC) were extracted from phone calls recorded with Motorola smartphones, achieving 92.3% classification accuracy. Although the study benefited from real-world data collection conditions, it focused exclusively on voice, omitting critical motor aspects like gait and posture. Suppa et al. [11] conducted a more detailed acoustic analysis using OpenSMILE software to extract 6,139 features from voice recordings of 115 PD patients and 108 healthy participants. The support vector machine (SVM)-based model demonstrated high diagnostic accuracy and additionally showed that L-Dopa therapy moderately improved speech symptoms. However, the model’s high feature dimensionality and lack of clinical interpretability posed challenges for real-world applications. He et al. [5] also used voice data from the MDVR-KCL and mPower datasets, applying preprocessing steps such as denoising and segmentation before extracting 446 acoustic features. The model achieved over 90% accuracy, demonstrating strong potential for smartphone-based digital clinical assessments. Still, like previous studies, it did not integrate other symptom modalities. While the use of both scripted and spontaneous speech improved generalizability, it also introduced standardization challenges, and the clinical relevance of many extracted features remained unexplained. Lim et al. [8], as mentioned, integrated voice with facial expressions, yet still lacked a comprehensive motor symptom evaluation.

### Posture and pose-based studies

Studies in this category aimed to quantify postural abnormalities—a common motor symptom in PD—using image-based or sensor-derived pose estimation techniques. Shin et al. [10] developed a deep learning algorithm to assess anterior flexion angle (AFA) and dropped head angle (DHA) from lateral profile photographs of PD patients. Their method achieved excellent agreement with manual labels, with intraclass correlation coefficients exceeding 0.95. However, the model focused only on static images, lacking the ability to capture dynamic variations in posture or its evolution over time. Additionally, other PD symptoms, such as voice or gait impairments, were not considered.

Zubiena et al. [3] utilized a dynamic posturography system integrated with wearable inertial sensors to assess postural control in 20 PD patients and 15 healthy controls. A total of 72 features were extracted and evaluated using 52 different ML classifiers. The highest classification accuracy of 0.95 was achieved by the k-Nearest Neighbors (KNN) model. However, the reliance on wearable IMU sensors presents notable limitations. Beyond the financial cost and technical setup requirements, the use of body-worn sensors may also pose challenges related to user comfort and long-term acceptability.

Zhang et al. [16] proposed a Kinect-based system that collected 3D joint data from 70 PD patients. These data were reduced to 2D trunk-related features and labeled according to MDS-UPDRS-III item 3.13 (related to posture), before being classified using a decision tree model. With an ICC of 0.940, the system showed strong agreement with clinical assessments, but it evaluated only static posture based on a single movement and did not analyze more complex dynamic tasks like turning or walking. Both studies highlight the potential of pose estimation for objective PD assessment but underscore the limitations of unimodal focus and restricted motor scope.

The review of existing studies reveals that the majority of AI-based models for PD detection are unimodal, focusing on a single symptom domain such as gait, facial expressions, or voice. Gait analysis, often relying on skeleton-based data or kinematic features, is the most commonly used modality; however, these models typically overlook other critical aspects of the disease. Similarly, several studies have explored facial expressions or voice impairments in isolation, with only a few attempting partial multimodal integration. Notably, Lim et al. [8] is the only study identified that combines both voice and facial expression data, yet it still excludes gait and postural abnormalities. Despite the promising performance of these individual models, their limited clinical scope reduces generalizability and fails to capture the multidimensional nature of PD. As illustrated in Table 1, most existing approaches remain confined to single-modality analysis, and no prior work has been found that integrates all four key clinical assessments—walking, facial expressions, voice, and posture—within a unified framework. This highlights a significant gap in the literature. The newly proposed framework distinguishes itself by simultaneously analyzing these four modalities. This not only enhances diagnostic performance but also provides modality-specific, interpretable outputs that contribute meaningfully to clinical decision support processes, thereby addressing the shortcomings of prior approaches and offering a more comprehensive assessment of PD.

**Table 1:** Comparison of state-of-the-art Parkinson’s disease detection models in the literature with the proposed framework

Reference	Walking	Face	Voice	Posture
Biase et al., (2022) [2]	$\checkmark$	-	-	-
Cesare et al., (2024) [4]	-	-	$\checkmark$	-
He et al., (2024) [5]	-	-	$\checkmark$	-
Huang et al., (2024) [7]	-	$\checkmark$	-	-
Zhang et al., (2021) [16]	-	-	-	$\checkmark$
Mehta et al., (2021) [9]	$\checkmark$	-	-	$\checkmark$
Shin et al., (2022) [10]	-	-	-	$\checkmark$
Zubiena et al., (2022) [3]	-	-	-	$\checkmark$
Suppa et al., (2022) [11]	-	-	$\checkmark$	-
Tian et al., (2024) [12]	$\checkmark$	-	-	-
Xu et al., (2023) [14]	-	$\checkmark$	-	-
Zeng et al., (2023) [15]	$\checkmark$	-	-	-
Zhao et al., (2024)[17]	$\checkmark$	-	-	-
Lim et al., (2022) [8]	-	$\checkmark$	$\checkmark$	-
**Our Study**	$\checkmark$	$\checkmark$	$\checkmark$	$\checkmark$

As clearly shown in Table 1, current research on the automatic diagnosis of Parkinson’s disease mostly focuses on a single symptom and evaluates these symptoms individually. Few studies examine multi-modal combinations; however, these approaches generally integrate at most two methods and often overlook important motor or postural features. Consequently, most current AI-based diagnostic systems remain limited to fragmented assessments and can not adequately represent the multi-modal clinical nature of PD. Differences in data collection protocols and method-specific modeling processes hinder the development of unified and clinically interpretable frameworks. In contrast, the proposed study addresses this gap by simultaneously analyzing walking, facial expression, voice, and posture within a single decision support architecture. By evaluating these four clinically relevant methods together, our framework expands on existing discoveries. This comprehensive integration provides a more holistic representation of Parkinson’s symptoms.

## Dataset generation

A collaborative study was conducted with the Department of Neurology at Kocaeli University to develop an AI-assisted diagnostic system for PD. The data collection protocol and decision-making system were designed through interdisciplinary consultation, incorporating clinical expertise to ensure methodological robustness and clinical relevance. All data collection processes were conducted under the supervision of a neurologist, a neurology resident, and six computer engineers as part of the research team. Ethical approval for the study was granted by the Ethical Committee for Non-Invasive Clinical Research at Kocaeli University (Decision No. KÜ GOKAEK-2024/13.09), as described in the Ethical Approval section.

As an initial step, a dedicated test room was established at the Simulation Center of Kocaeli University Training and Research Hospital. This room was designed to accommodate both Parkinson’s patients and healthy control participants, providing sufficient room for natural walking and maneuver execution. To facilitate the evaluation of gait dynamics and turning behaviors, an “L”-shaped walking track was implemented.

**Fig. 1:**
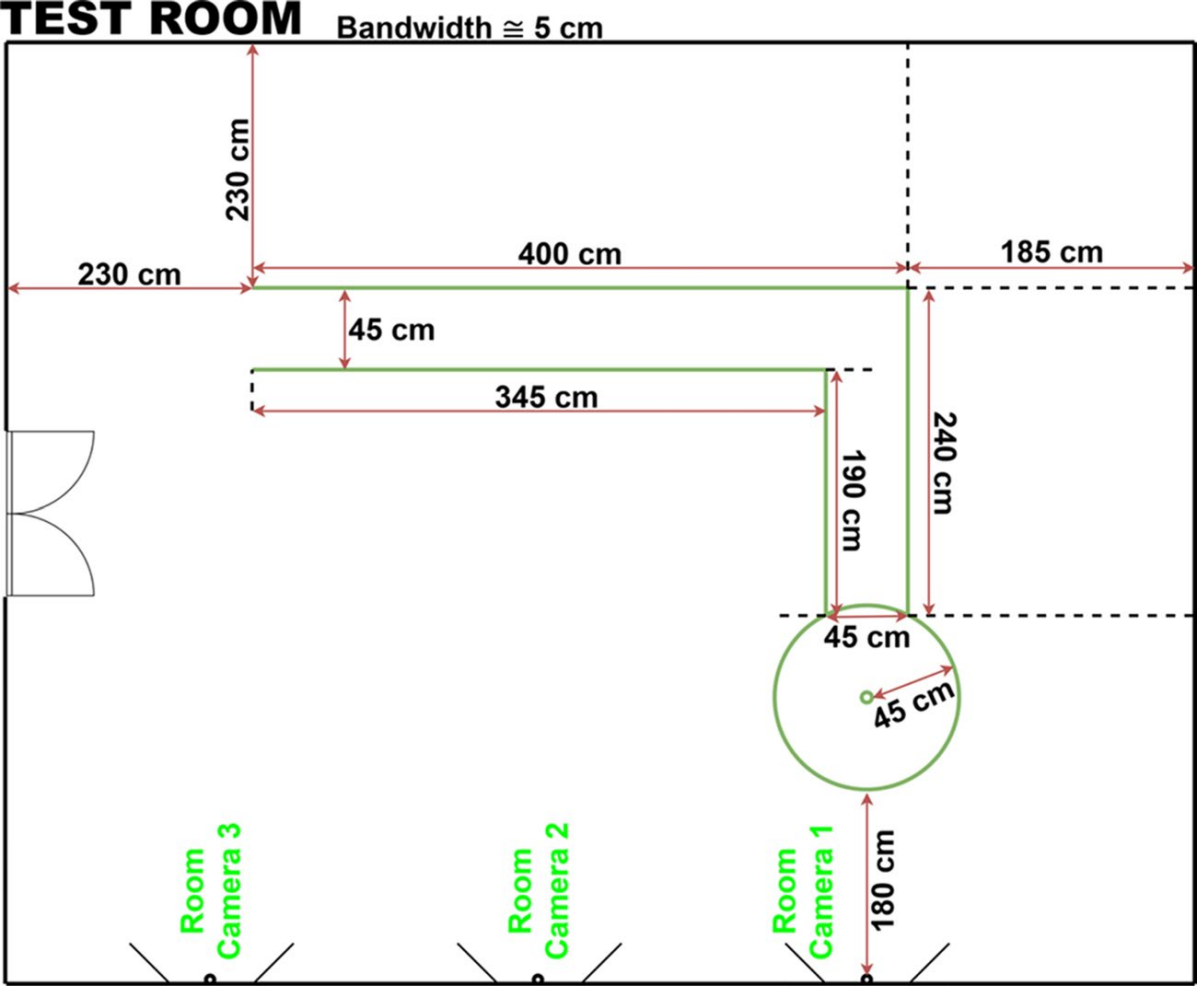
Technical schematic of the test chamber setup used for recording walking videos from Parkinson’s patients and healthy controls as part of the gait analysis module

For the development of the walking analysis component, referred to as the “Walking Modality Module”, walking videos were recorded from both Parkinson’s patients and healthy control participants. To capture movements from multiple perspectives, three fixed cameras were mounted on the walls at a height of approximately 1.85 meters. In order to reduce visual noise and improve the quality of the recordings, visual separators were positioned directly opposite each camera. The technical layout and configuration of the test environment are presented in Fig. 1, ensuring transparency and reproducibility in the data acquisition methodology.

**Fig. 2:**
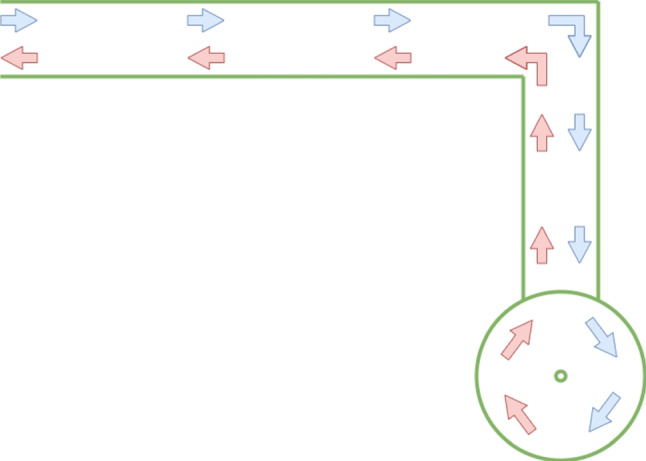
L-shaped walking track used for walking data collection

During data collection, participants were instructed to walk naturally and freely along the L-shaped walking track, taking care not to step directly on the tape lines that marked the path boundaries. The intended gait pattern is illustrated in Fig. 2.

**Fig. 3:**
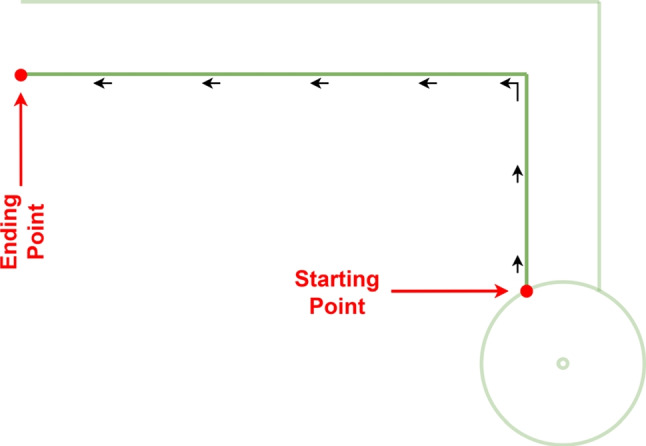
The line followed during the tandem walking task

After recording participants’ natural walking behavior using the fixed room cameras, each individual was asked to perform a tandem walking task along a designated line within the L-shaped walking track. These tandem walking sequences were also recorded using the same camera setup. The intended path for this task is illustrated in Fig. 3. The L-shaped walking track was deliberately selected to capture multiple clinically relevant gait characteristics within a single, natural walking sequence. Specifically, this configuration allows participants to perform a directional turn, approach the wall-mounted room camera, and subsequently move away from it while completing a straight walking segment. As a result, the camera labeled Room Camera 1 in Fig. 1 acquires both frontal and posterior profile views of the participant at different phases of the same trial, which is not achievable with a purely linear configuration.

Following the gait recordings, the data collection process proceeded with the acquisition of postural and facial expression data, which were used to develop the posture and face modules of the diagnostic system. For this phase, participants stood against the plain white background wall of the test room and were guided through a sequence of simple posture-related commands. At five-second intervals, participants were instructed to turn to the right, turn to the left, lift one foot, and then lift the other. These actions were captured in approximately 20-second videos using a Samsung Galaxy A51 smartphone camera. The raw video footage was used to train the posture module, while specific video frames—particularly those in which participants looked directly at the camera—were extracted to build the face module.

**Fig. 4:**
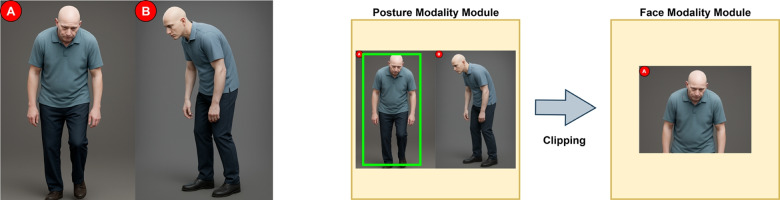
Collection of postural stance data and extraction of facial frames for the face module dataset

Figure 4 illustrates this dual-purpose video capture approach, highlighting how both postural features commonly observed in advanced PD and facial expressions were utilized for separate but related diagnostic objectives.

To develop the final component of the diagnostic assistant—the voice module—participants were instructed to perform a structured speech task in Turkish. (1) reciting the sentence “The Great Leader Mustafa Kemal Atatürk founded the Republic of Türkiye,” (2) counting aloud from one to twenty, and (3) reciting the days of the week in order. All voice recordings were captured using a Sony ICD-PX470 digital voice recorder to ensure high-quality audio suitable for subsequent acoustic feature extraction and analysis.

Table 2 presents the number of Parkinson’s patients and healthy individuals recorded for each modality in the dataset. The walking and tandem walking modalities were built using data from 21 individuals diagnosed with PD and 20 healthy controls. For the face modality, data from 38 patients with PD and 21 healthy individuals were used. The voice modality included observations from 33 Parkinson’s patients and 21 healthy controls. Lastly, the posture modality was developed using data from 37 Parkinson’s patients and 20 healthy individuals. As shown in Table 2, the number of Parkinson’s patients and healthy individuals observed in the clinic and included in the dataset varies across modalities. Although the sample sizes differ, the datasets largely comprise the same participants. The smaller number of patients in the walking and tandem walking modalities is due to the inability to obtain video recordings from some patients, resulting from PD progression. Similarly, the reduced size of the audio dataset reflects the exclusion of low-quality recordings. For each modality, the maximum number of high-quality data samples was retained. Ultimately, all modalities were equally weighted, based on expert physician guidance, and combined to produce the final PD diagnosis.

**Table 2:** Number of observations for Parkinson’s and healthy classes across each modality

Modality	Parkinson	Healthy
Walking Modality	21	20
Tandem Walking Modality	21	20
Face Modality	38	21
Voice Modality	33	21
Posture Modality	37	20

In conclusion, the dataset that forms the foundation of the proposed multimodal diagnostic support system was carefully curated. The collection process was designed to comprehensively capture key motor and vocal symptoms of PD across four distinct modalities: gait, posture, facial expression, and voice. Conducted within a controlled test environment and informed by clinical expertise, this protocol ensures both scientific rigor and clinical applicability. As such, the resulting dataset provides a valuable resource for training and validating future AI-based models for the digital assessment of PD.

## Framework modules and experimental results

Our proposed framework consists of four main modules: the walking modality module, the face modality module, the voice modality module, and the posture modality module. In this section, we provide a detailed overview of these modules and evaluate their performance in diagnosing idiopathic Parkinson’s disease.

### Walking modality module

We evaluated the walking modality module through two main processes: keypoint extraction and modeling, as detailed below.

#### Keypoint extraction

**Fig. 5:**
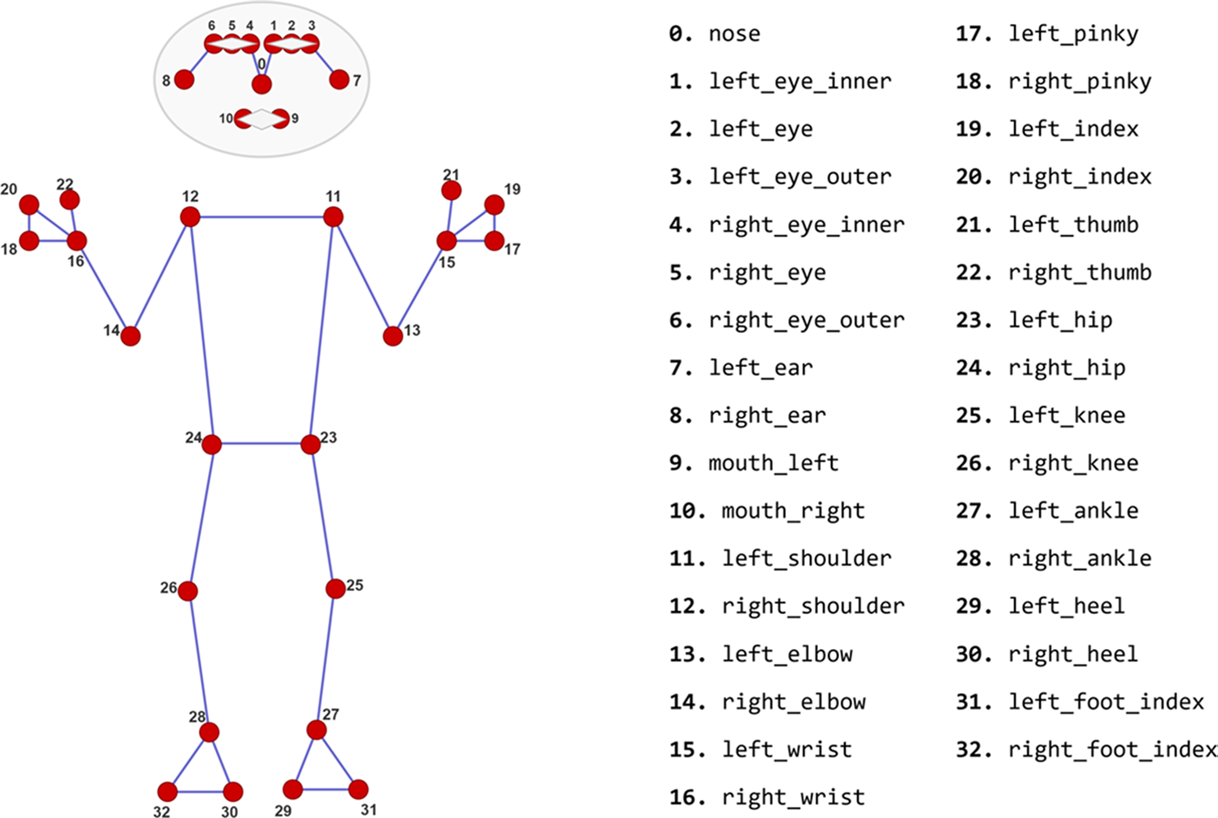
The set of anatomical keypoints identified by the MediaPipe library [6]

To develop the walking modality module, an L-shaped walking track was utilized to comprehensively evaluate participants’ natural walking and turning abilities. Participants first walked freely along the L-shaped path, followed by a tandem walk on the same route. Both walking styles were video-recorded, and body joint angles were extracted using the MediaPipe library—a robust pose estimation framework capable of identifying 33 anatomical keypoints across the head, face, upper body, lower body, and chest regions, as shown in Fig. 5. These keypoints were used to calculate various angles and distances through trigonometric and vector-based methods. For instance, combinations of two or three keypoints (e.g., hip, knee, ankle) were used to compute joint angles, enabling a detailed biomechanical analysis. MediaPipe’s provision of both 2D and 3D coordinates further facilitated multi-dimensional gait evaluation.

Using keypoints such as the left hip, right hip, left knee, right knee, left ankle, right ankle, left heel, right heel, left toe, and right toe, gait data from both walking styles (natural and tandem walking) were analyzed using cosine and Euclidean similarity metrics, as presented in Eqs. 1 and 2, respectively.

1 $$\begin{aligned} \cos (\theta _{\alpha }) = \frac{\boldsymbol{\alpha }_i \cdot \boldsymbol{\alpha }_j}{\Vert \boldsymbol{\alpha }_i\Vert \, \Vert \boldsymbol{\alpha }_j\Vert } \end{aligned}$$

where $\boldsymbol{\alpha }_i$ and $\boldsymbol{\alpha }_j$ represent the time series vectors of a specific joint angle or spatial gait feature (e.g., right knee flexion, hip displacement, heel trajectory), extracted from different walking sessions or participants. This formulation quantifies the temporal similarity between two signals, offering a metric to assess consistency or deviation in movement patterns.

2 $$\begin{aligned} d_{\text {keypoint}}^{(t)} = \sqrt{(x_{k,t+1} - x_{k,t})^2 + (y_{k,t+1} - y_{k,t})^2 + (z_{k,t+1} - z_{k,t})^2} \end{aligned}$$

where $(x_{k,t}, y_{k,t}, z_{k,t})$ and $(x_{k,t+1}, y_{k,t+1}, z_{k,t+1})$ denote the 3D coordinates of keypoint *k* at time frames *t* and $t+1$, respectively. The keypoint *k* can refer to any anatomical landmark such as the right heel, left hip, or right toe. This generalized distance function enables the calculation of temporal displacements for various body parts in gait analysis.

**Fig. 6:**
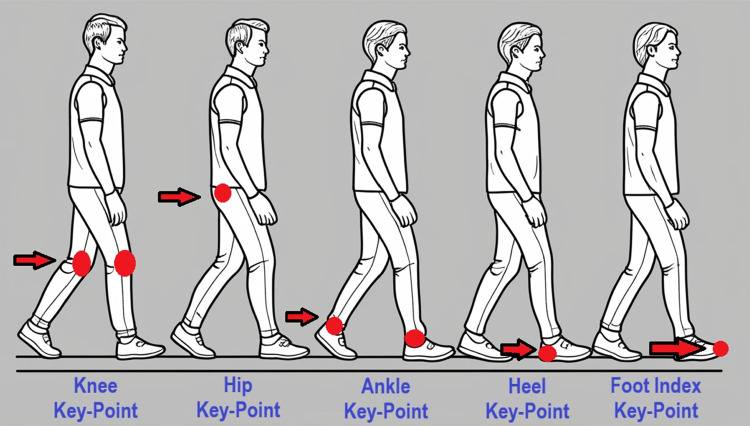
Visualization of the critical keypoints used in the analysis of natural and tandem walking

To differentiate between individuals with PD and healthy controls, the following angles were computed: right/left foot lift heights, 2D/3D foot placement angles (right/left), knee flexion angles (right/left), 2D/3D hip horizontal displacement angles, and 2D/3D heel horizontal displacement angles. Among these, hip and heel horizontal displacement angles are particularly important for assessing gait stability and motor control. The hip angle captures lateral displacement of the hip in the horizontal plane, while the heel angle indicates lateral heel movement—both of which are often elevated in Parkinsonian gait. The foot placement angle refers to the orientation between the foot and the ground during a step. Abnormal angles, such as inward or outward rotation, are typical in PD and can increase fall risk and disrupt symmetry. Similarly, the knee flexion angle, which reflects knee bending during gait, is often reduced in individuals with Parkinson’s, contributing to a stiffer and less efficient walking pattern. The critical keypoints involved in analyzing natural and tandem walking are shown in Fig. 6. Based on the time each participant took to complete the L-shaped walking track during both natural and tandem walking trials, the resulting variable-length time series were transformed into fixed-length vectors of 1200 dimensions using a sliding-window approach. Each participant was assigned an index, and the corresponding angle values were stored in a MongoDB database.

#### Modeling

**Fig. 7:**
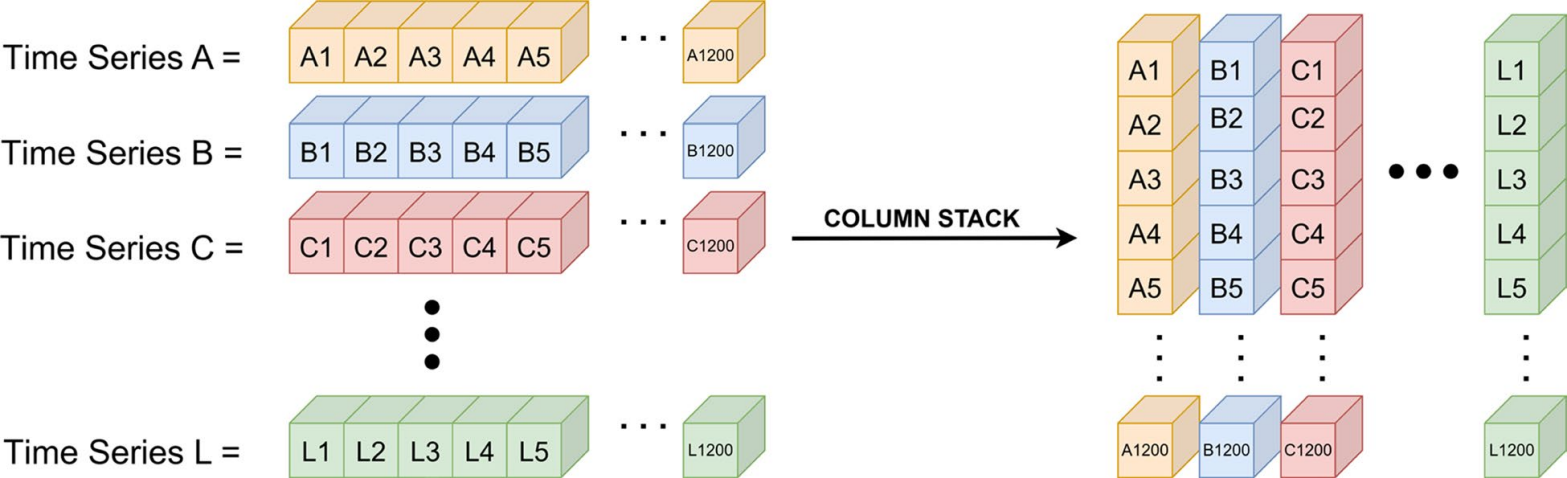
Column stack method for time series structuring

All time series lists stored in each record of the MongoDB database were retrieved and transformed into a two-dimensional array using the NumPy library. During this transformation, each time series feature was concatenated column-wise, resulting in a matrix with dimensions (TimeSteps $\times$ NumFeatures), achieved using the column stack function. This structure was then formatted to serve as input for the Gated Recurrent Unit (GRU) model. The column stacking process is illustrated in Fig. 7.

As illustrated in Fig. 7, twelve distinct types of temporal information extracted from all video frames are visualized. Each label from A to L corresponds to a specific temporal parameter, including right and left foot lift heights; right and left foot placement angles in 2D and 3D; right and left knee flexion angles; hip horizontal displacement angles in 2D and 3D; and heel horizontal displacement angles in 2D and 3D.

Once the data were appropriately formatted, several data augmentation techniques were applied to the time series, including noise addition, acceleration, deceleration, reversal, and shifting. In cases where these operations altered the sequence lengths, linear interpolation was used to normalize all sequences to a uniform length of 1200 time steps. Finally, standard scaling was applied across all features.

**Fig. 8:**
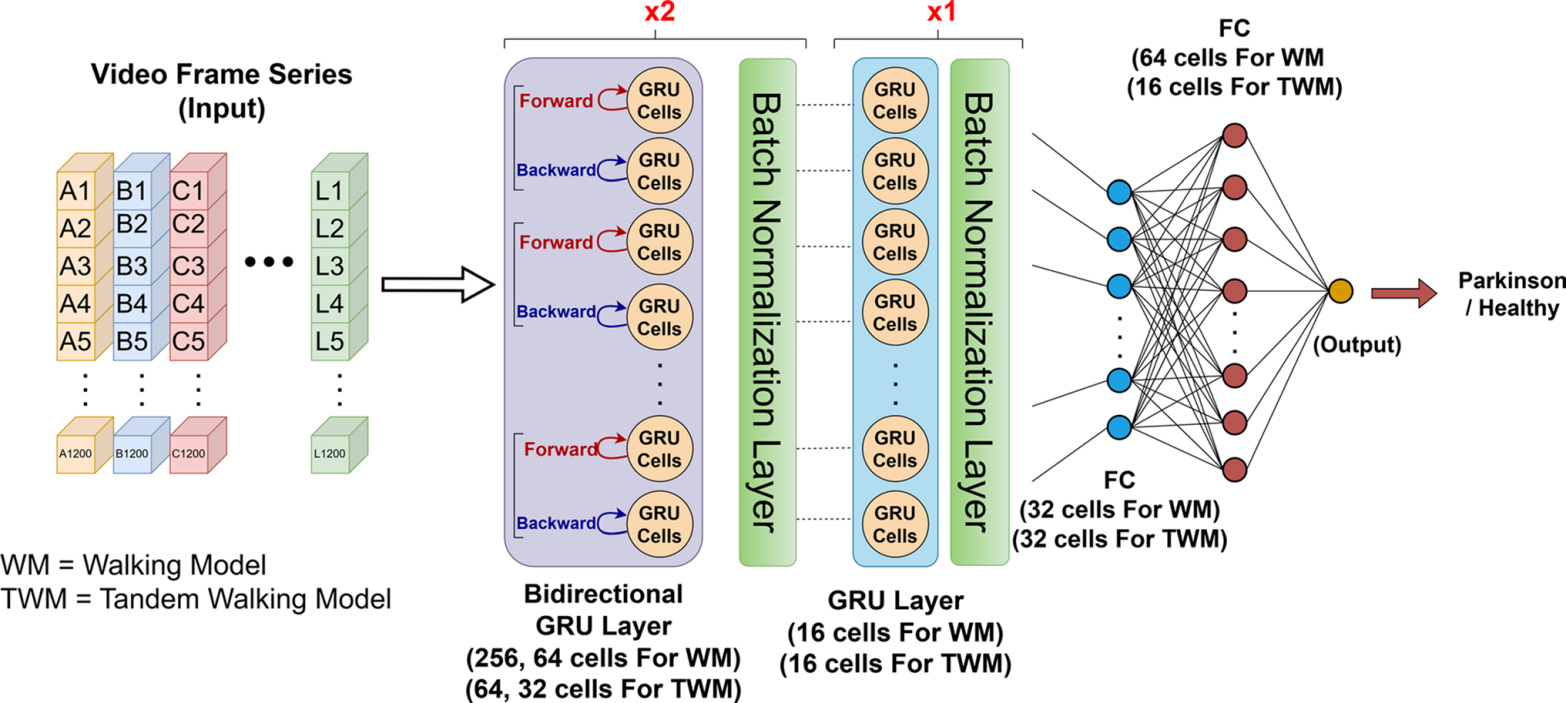
Walking modality model architecture

For the walking modality, 41 original observations—21 from Parkinson’s patients and 20 from healthy controls—were augmented using four different data augmentation techniques, resulting in a total of 205 observations. These augmented observations were used for model training and performance evaluation. For the modeling phase, a GRU-based architecture grounded in Recurrent Neural Network (RNN) principles was developed. To enable bidirectional temporal feature extraction, bidirectional GRU layers were employed, followed by dense layers to facilitate classification of the learned features. Based on this architecture, two distinct models were trained: one using natural walking data and the other using tandem walking data. The model architecture for both scenarios is illustrated in Fig. 8.

Following model construction, hyperparameter optimization was conducted to ensure efficient training. A random search method was used to evaluate various combinations of learning rate, number of layers, GRU units, dropout rate, and batch size. Each configuration was assessed based on training accuracy and validation loss, and the best-performing parameter set was selected for the final model. The search space for hyperparameter tuning is provided in Table 3.

**Table 3:** Hyperparameter search space for bidirectional GRU model

Model	Hyperparameter	Possible Values
Bidirectional GRU	GRU Units 1	[64, 128, 256]
	GRU Units 2	[32, 64, 128]
	GRU Units 3	[16, 32, 64]
	Dense Units 1	[32, 64, 128]
	Dense Units 2	[16, 32, 64]
	Dropout Rate 1	[0.3, 0.4, 0.5]
	Dropout Rate 2	[0.3, 0.4, 0.5]
	Dropout Rate 3	[0.2, 0.3, 0.4]
	Dropout Rate 4	[0.1, 0.2, 0.3]
	Dropout Rate 5	[0.1, 0.2, 0.3]
	Learning Rate	[0.0001, 0.0005, 0.001]
	Optimizer	[rmsprop, adam]
	Batch Size	[8, 16, 32]
	Epochs	[50, 75, 100]

To assess model generalizability, Stratified K-Fold Cross-Validation was applied, accounting for class imbalances. The dataset was divided into *K* equally sized subsets, with class distributions preserved in each fold. In each iteration, one subset was used for validation, while the remaining $K-1$ subsets were used for training. This method enabled robust evaluation across multiple data partitions and reduced the risk of overfitting. As a result of this methodology, the final model achieved high classification accuracy and low error rates on gait data. The same methodology was applied to tandem walking data, yielding a second model trained using identical procedures. Using a random search strategy, 20 different hyperparameter combinations were generated and evaluated using 5-fold stratified cross-validation. For the walking modality, the optimal combination was identified as follows: GRU Units 1: 256, GRU Units 2: 64, GRU Units 3: 16, Dense Units 1: 32, Dense Units 2: 64, Dropout Rates: 0.4, 0.4, 0.3, 0.1, 0.1, Learning Rate: 0.0005, Optimizer: RMSprop, Epochs: 75. Similarly, for the tandem walking modality, 20 combinations were evaluated using the same strategy, and the optimal configuration was determined as: GRU Units 1: 64, GRU Units 2: 32, GRU Units 3: 16, Dense Units 1: 32, Dense Units 2: 16, Dropout Rates: 0.5, 0.5, 0.2, 0.3, 0.1, Learning Rate: 0.001, Optimizer: Adam, Epochs: 75. The performance metrics of the final models trained with these optimal hyperparameters are presented in Table 4. As reported in the table, the walking modality model demonstrates consistently higher classification performance compared to the tandem walking model across all evaluation metrics. This performance gain can be attributed to the fact that natural walking better preserves individual gait characteristics, allowing Parkinsonian motor impairments to emerge more clearly.

**Table 4:** Evaluation of walking and tandem walking modality models’ classification performance

Model	Accuracy	Precision	Recall	$F_1$-Score	ROC-AUC
Walking Modality Model	0.9268	0.9339	0.9238	0.9274	0.9786
Tandem Walking Modality Model	0.8780	0.8931	0.8761	0.8808	0.9423

### Face modality module

We evaluated the face modality module through two main processes: action unit extraction and modeling, as detailed below.

#### Action unit extraction

During the development of the face modality module, data reflecting responses in the cephalocervical region were collected via a mobile device, as detailed in Section 3, based on participants’ reactions to specific commands. A total of 59 Facial Action Units (AUs) were extracted using the Py-Feat library from these video recordings. These AUs were used to examine motor dysfunctions in facial expressions, particularly hypomimia (reduced facial expressiveness), which is commonly observed in individuals with PD. The aim of this analysis was to detect various conditions—such as blink frequency, hypomimia, oromandibular dystonia, jaw tremor, and facial asymmetry—by comparing the facial data of Parkinson’s patients and healthy individuals. Using the Py-Feat library, we specifically analyzed facial muscle groups identified in the literature as being associated with PD. Based on these groups and the observed participant data, we engineered new features that capture the relationships between specific Action Units (AUs). As part of this process, we formulated 12 mathematical equations (Eqs. 4 to 15), which were integrated into our classification model. The variables denoted by *p* in these equations represent parameter combinations that yielded the highest accuracy in the logistic regression model, as detailed in Eq. 3. Additionally, the variable *time* refers to the total duration of each video file.

3 $$\begin{aligned} (p_0,p_1,p_2)\in \{1,\dots ,10\} \end{aligned}$$

4 $$\begin{aligned} \text {Perioral And Periorbital Expression Reduction} = \frac{(\text {AU06}p_0 + \text {AU09}p_0 + \text {AU10}p_0 + \text {AU11}p_0 + \text {AU12}p_0 + \text {AU17}p_0)}{p_2 \cdot p_1} \end{aligned}$$

The equation for ’Perioral and Periorbital Expression Reduction’ incorporates action units (AUs) AU06, AU09, AU10, AU11, AU12, and AU17. These units capture limitations in blinking, smiling, and cheek movements, which are key indicators of diminished muscle activity in the peripheral facial regions (Eq. 4).

5 $$\begin{aligned} \text {Lower-Facial Mimic Weakness And Rigidity} = \frac{p_0(\text {AU06} + \text {AU12}) - p_1(\text {AU25} + \text {AU26})}{p_2} \end{aligned}$$

Similarly, “Lower-Facial Mimic Weakness” is quantified through a combination of AU06 and AU12 (targeting the Orbicularis oculi and Zygomaticus major), along with AU25 and AU26 (associated with the Depressor Labii Inferioris and Masseter), effectively measuring the decline in muscle activity across the periorbital and perioral zones. This approach highlights the weakening of lower facial muscles and increased rigidity between the chin and lips (Eq. 5).

6 $$\begin{aligned} \text {Dystonia Oromandibularis} = (\text {AU07}p_0 + \text {AU10}p_1 + \text {AU11}p_2) + (\text {AU24}p_2) \end{aligned}$$

To evaluate “Dystonia Oromandibularis,” the model utilizes AU07, AU10, AU11, and AU24, which collectively reflect abnormal muscle function around the nasolabial groove and upper lip (Eq. 6).

7 $$\begin{aligned} \text {Hypomimia Mentis} = \frac{\text {AU12}p_0 + \text {AU17}p_1 + \text {AU24}p_2}{3} \end{aligned}$$

The symptom of “Hypomimia Mentis” is assessed through reductions in lip pressing and jaw motion, using AU12, AU17, and AU24 to indicate decreased expressivity in the mouth and jaw (Eq. 7).

8 $$\begin{aligned} \text {Hypomobilitas Oris et Mentis} = \frac{\text {AU12} + p_1 p_2 \cdot \text {AU17} + p_1 p_2 \cdot \text {AU24}}{3} \end{aligned}$$

For “Hypomobilitas Oris et Mentis,” the equation evaluates limitations in lip and jaw movements by emphasizing AU12, AU17, and AU24—with added weighting on AU17 and AU24—to isolate Parkinsonian facial rigidity from emotional mimic activity (Eq. 8).

9 $$\begin{aligned} \text {Facies Hypomimica} = \frac{\text {AU06} + \text {AU07} + \text {AU09} + \text {AU10} + \text {AU11} + \text {AU12} + \text {AU14} + \text {AU15} + \text {AU17} + \text {AU23} + \text {AU24} + \text {AU28} + \text {AU43}}{13 \cdot \text {time}} \end{aligned}$$

The broader condition of “Facies Hypomimica” is addressed in Eq. 9, which combines AU06, AU07, AU09, AU10, AU11, AU12, AU14, AU15, AU17, AU23, AU24, AU28, and AU43 to comprehensively measure the generalized reduction in facial expressiveness and motor function characteristic of PD.

10 $$\begin{aligned} \text {Amplitudo Mimica Decrescens} = \left( \frac{1}{1+\text {time}}\right) \left( \text {AU06} + \frac{\text {AU12}}{p_0}\right) \left( -(\text {AU25} + \frac{\text {AU26}}{p_0})\right) \end{aligned}$$

To capture reductions in the amplitude of mimic movements, the “Amplitudo Mimica Decrescens” (Eq. 10) evaluates AU06, AU12, AU25, and AU26 alongside a temporal component, focusing on the periorbital and oral regions.

11 $$\begin{aligned} \text {Atrophia Musculorum Palpebrarum} = 1 - \frac{\text {AU06} + \text {AU07} + \text {AU43}}{p_2} \end{aligned}$$

Deterioration in eyelid motion, reflecting weakening of the orbicularis oculi and levator palpebrae superioris muscles, is assessed through the “Atrophia Musculorum Palpebrarum” (Eq. 11) using AU06, AU07, and AU43.

12 $$\begin{aligned} \text {Atrophia Musculorum Oris et Mentis} = 1 - \left( p_2((\text {AU17} + \text {AU24})p_0) - p_1\text {AU28}\right) \end{aligned}$$

Likewise, atrophy in the lip and jaw region—signaled by oromandibular dystonia and reduced fine motor control—is measured via AU17, AU24, and AU28 in the “Atrophia Musculorum Oris et Mentis” equation (Eq. 12).

13 $$\begin{aligned} \text {Dysfunctio Synchroniae Lineae Medianae} = 1 - \left( 0.3 p_1 \cdot \frac{\text {AU04}+\text {AU07}}{2} + 0.2 p_1 \cdot \frac{\text {AU10}+\text {AU17}}{2}\right) \end{aligned}$$

Coordination deficits among the eyebrows, eyes, upper lip, and chin are captured by the “Dysfunctio Synchroniae Lineae Medianae” (Eq. 13), employing AU04, AU07, AU10, and AU17 to expose impaired midline synchronization.

14 $$\begin{aligned} \text {Dysfunctio Aperturae Palpebrarum} = 1 - (p_1 \cdot p_2 \cdot \text {AU43}) \end{aligned}$$

The “Dysfunctio Aperturae Palpebrarum” (Eq. 14) isolates periorbital expression loss due to blepharospasm and muscle rigidity using AU43, thereby revealing disorders in eyelid dynamics.

15 $$\begin{aligned} \text {Hypomimia et Dyskinesia Oromandibularis et Ocularis} = \frac{\text {AU05} + \text {AU25} + \text {AU26} + \text {nf7} + \text {nf8} + \text {nf11} + \text {nf13} + \text {nf14}}{13 \cdot \text {time}} \end{aligned}$$

Finally, “Hypomimia et Dyskinesia Oromandibularis et Ocularis” (Eq. 15) integrates AU05, AU25, AU26, and features nf7, nf8, nf11, nf13, and nf14— corresponding respectively to Eqs. 11, 12, 10, 13, and 14—to comprehensively assess mimic impairments in both the lower face and periorbital regions.

#### Modeling

A structured CSV file was created to include both extracted facial action units and newly derived Parkinsonism-related characteristics, along with participant registration information. Various data augmentation techniques were applied to the constructed data structure, primarily involving the addition of Gaussian noise and the use of SMOTE (Synthetic Minority Oversampling Technique). These operations aimed to address the class imbalance between people with Parkinson’s disease and healthy controls, support a more robust training process, and improve the generalization performance of the model on our relatively small dataset. In the next step, features were categorized into two groups: those with a high number of outliers and those with few outliers. Features with many outliers were further divided into positively and negatively correlated groups based on their correlation with the target variable. The Median Absolute Deviation (MAD) method was then applied to both groups to mitigate the influence of extreme values by averaging the absolute deviations from the median, which offers a more robust alternative to standard deviation-based methods. Following these operations, a refined data structure was obtained, in which the influence of outliers had been substantially reduced. In addition, several outlier removal techniques were applied to the combined dataset, which now also included the initially separated features with few outliers. Among these, the most effective approach was found to be the identification of outliers using the Interquartile Range (IQR) method, followed by replacing those values with the column mean. The IQR method was preferred due to its ability to effectively eliminate extreme values while preserving the overall distributional characteristics of the data. These outlier reduction steps were designed to improve the model’s decision-making accuracy in the presence of potential recording artifacts or spontaneous facial behaviors, such as sudden blinks, expressions of surprise, or nervous reactions.

**Fig. 9:**
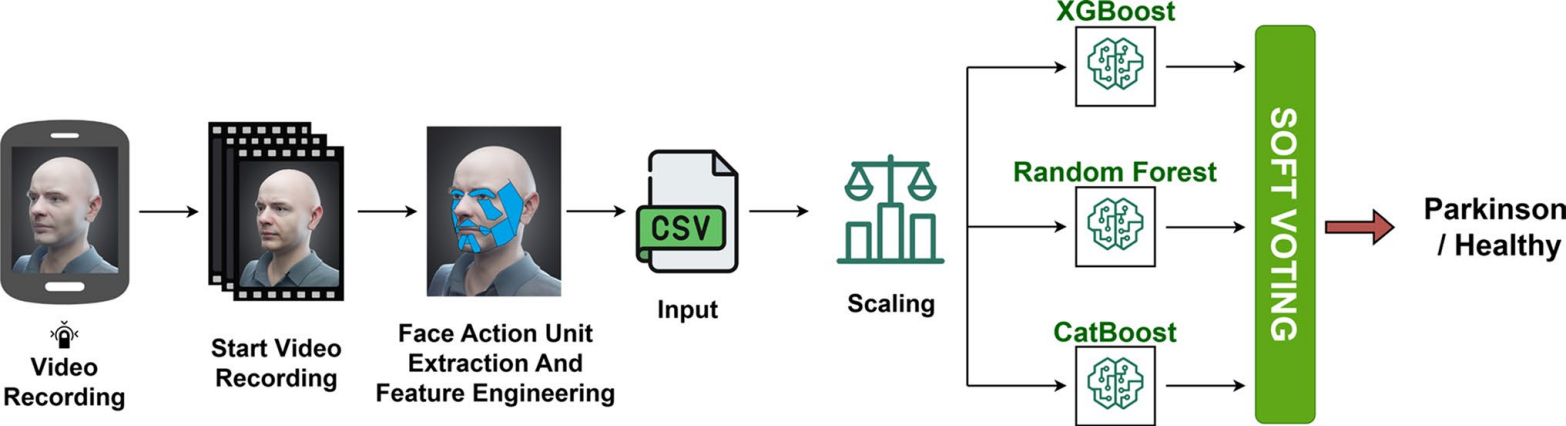
Face modality model architecture

In the modeling process, we utilized 12 equations derived from the features described in Section 4.2.1 (Eqs. 4–15). The dataset was divided into the training and testing subsets, 90% and 10%, comprising a total of 136 observations. For the modeling architecture, an ensemble architecture was developed, integrating three different tree-based classifiers: XGBoost, RF, and CatBoost. These models were combined using a soft voting strategy to leverage the individual strengths of each classifier. The architecture of the proposed ensemble model is illustrated in Fig. 9.

Following the development of the model architecture, hyperparameter optimization was conducted to ensure the most efficient training process. This involved systematically testing various combinations of key parameters—such as learning rate, number of trees, and model depth—using the GridSearchCV method. Each parameter set was evaluated based on both training performance and validation loss. The optimal configuration was selected as the one yielding the highest accuracy and lowest loss. The complete hyperparameter search space utilized for this optimization is presented in Table 5.

**Table 5:** Hyperparameter search space for face module

Model	Hyperparameter	Possible Values
XGBoost	number of estimators	[30, 50, 90]
	learning rate	[0.001, 0.005, 0.01]
	maximum depth	[2, 3, 7]
RF	number of estimators	[30, 50, 80]
	maximum depth	[3, 5, 8]
CatBoost	number of iterations	[200, 250, 350]
	tree depth	[5, 6, 8]
	learning rate	[0.001, 0.005, 0.01]

To objectively assess the generalization performance of the model, we employed Stratified K-Fold Cross-Validation with $K=3$. This approach helps mitigate the effects of class imbalance by preserving the distribution of classes across all folds. The performance metrics of the facial expression model, optimized with the best hyperparameters identified through this three-fold evaluation, are given in Table 6. As shown in the table, the facial modality model achieves high and balanced classification performance; this is reflected in the strong $F_1$ score and ROC-AUC value obtained in 38 Parkinson’s patients and 21 healthy individuals. This result demonstrates that engineering-based facial action unit features effectively capture facial motor impairments associated with Parkinson’s disease and supports the suitability of facial expression analysis as a complementary diagnostic method.

**Table 6:** Evaluation of face model classification performance

Model	Accuracy	Precision	Recall	$F_1$-Score	ROC-AUC
Face Modality Model	0.9286	1.0000	0.8571	0.9231	0.9796

### Voice modality module

We evaluated the voice modality module through two main processes: feature extraction unit and modeling, as detailed below.

#### Feature extraction

During the development of the voice module, three distinct speech tasks were administered to the participants to evaluate the characteristic vocal symptoms of Parkinson’s disease, as detailed in Section 3. These tasks facilitated the collection of both structured and spontaneous speech samples. The feature extraction process involved voice acquisition, digital signal conversion, and subsequent extraction of relevant acoustic features for analysis. The collected voice data were analyzed using digital signal processing techniques. A total of 90 features were extracted from each audio recording using the Librosa library within a Python environment. All audio samples were standardized to a fixed sampling rate of 44.1 kHz, chosen for its ability to accurately capture the natural frequency range of human speech and maintain consistency with previous studies. The extracted features encompassed both structural and dynamic aspects of speech in the time and frequency domains, serving as meaningful indicators of Parkinson’s-related vocal impairments. A detailed list of these features and their corresponding functions is provided in Table 7. Note that the “Count” column indicates the number of features; for example, there are 12 features representing Chrome STFT.

**Table 7:** Overview of the extracted voice features

Feature	Count	Description
Zero Crossing Rate (ZCR)	1	Measures how often a speech signal crosses the zero line. In other words, it counts the number of times the signal transitions between positive and negative amplitude values.
Chroma STFT	12	Chroma features represent the energy distribution across the 12 chromatic notes (C, C#, D, etc.) in the audio spectrum. They are commonly used to analyze the melodic and harmonic characteristics of speech
MFCC Mean	20	MFCCs (Mel-Frequency Cepstral Coefficients) are features that represent the spectral characteristics of an audio signal.
MFCC Delta	20	Measures the rate of change (derivative) of MFCC features over time, offering insight into how rapidly the spectral characteristics evolve.
MFCC Delta2	20	The second derivative of the MFCC delta measures the acceleration of changes in MFCC features and provides information about the signal’s dynamics.
Root Mean Square Energy (RMSE)	1	Measures the signal’s energy by calculating the average of the squared amplitude values.
Spectral Centroid	1	Represents the spectral centroid, indicating the average frequency position or “center of mass” of the spectrum in the signal.
Spectral Bandwidth	1	Measures the spectral bandwidth, reflecting the spread of spectral energy across the frequency range.
Spectral Contrast	7	Measures the energy differences across frequency bands, helping to differentiate between high- and low-energy regions in the spectrum.
Spectral Roll-off	1	Defines the frequency below which a specified percentage of the total spectral energy is contained, indicating the amount of high-frequency content in the signal.
Tonnetz	6	Extracts relationships between tones and chords in the signal, enabling deeper analysis of the audio’s harmonic content.

Using the extracted features, our goal was to identify vocal impairments commonly associated with PD, such as monotonic speech, reduced energy, spectral shifts, and weakened prosody. MFCC and its derivatives (Delta and Delta2) captured temporal variations in the speech signal, allowing assessment of monotonicity. RMSE reflected speech intensity, while spectral features like centroid, bandwidth, and roll-off helped detect shifts in frequency distribution. Additionally, Tonnetz and Chroma features were used to analyze potential impairments in intonation and harmonic structure. The feature extraction process was applied to four different types of voice recordings per individual. For each participant, we created the following distinct data scenarios:

- **Sentence:** Recordings containing only the sentence recitation task (The Great Leader Mustafa Kemal Atatürk founded the Republic of Türkiye).
- **Counting:** Recordings containing only the task of counting aloud from one to twenty.
- **Days:** Recordings containing only the recitation of the days of the week.
- **Combined:** A single merged recording including all three tasks above in sequence.

We analyzed the audio recordings separately for each scenario, creating four distinct datasets. Each dataset includes feature vectors for individual participants, along with labels indicating the presence or absence of PD. This multi-scenario evaluation approach enables us to determine which type of speech content most effectively impacts diagnostic performance during modeling. To better understand the distribution of voice characteristics across classes, we analyzed the extracted acoustic features from both Parkinson’s patients and healthy individuals. This analysis revealed several distinct differences—particularly in spectral, tonal, and frequency-based components—that suggest a systematic separation between the two groups. In general, recordings from individuals with PD tend to exhibit higher average values in these features. These structural differences indicate that the extracted features capture meaningful patterns, reinforcing the potential of voice data as a valuable clinical assessment for PD diagnosis.

#### Modeling

Due to the limited sample size of the dataset used in the voice module, several preprocessing steps were applied before modeling. These included checking for missing values, statistical summarization, and feature standardization. The final dataset comprised voice recordings from 35 individuals diagnosed with PD and 21 healthy participants, resulting in a total of 56 observations. From each recording, 90 acoustic features were extracted using the Librosa library, capturing both spectral and prosodic characteristics relevant to Parkinsonian vocal symptoms. Before modeling, the dataset was stratified and split into 80% training and 20% test sets to ensure balanced class distributions. To increase data variability and improve model robustness against minor real-world variations, small-scale Gaussian noise was added to the existing voice features. This augmentation supports more generalized learning, especially under data-scarce conditions. To address class imbalance caused by the lower number of individuals with PD, the SMOTE method was employed to generate synthetic samples for the minority class. During modeling, three core ML algorithms were selected: K-Nearest Neighbors, Logistic Regression, and RF. Hyperparameter tuning was performed for each algorithm. The defined search spaces and parameter values are detailed in Table 8.

**Table 8:** Hyperparameter search space for voice module

Model	Hyperparameter	Possible Values
RF	n_estimators	[50, 100, 200]
	max_depth	[None, 10, 20, 30]
	min_samples_split	[2, 5, 10]
	min_samples_leaf	[1, 2, 4]
K-Nearest Neighbors	n_neighbors	[3, 5, 7, 9, 11, 13, 15]
	weights	[“uniform”, “distance”]
	metric	[“euclidean”, “manhattan”, “minkowski”]
Logistic Regression	C	[0.01, 0.1, 1, 10, 100]
	solver	[“liblinear”, “lbfgs”, “saga”]

We applied Stratified K-Fold Cross-Validation (K=5) to evaluate the models. This method preserved the proportional distribution of each class within every fold, enabling an objective assessment of model performance across different data subsets. Additionally, to evaluate final model performance on an independent dataset, 20% of the data was set aside as a test set. This split was also stratified to maintain class balance within the test set. The test data were excluded from both training and hyperparameter tuning and were used solely at the final stage to assess generalization performance. This approach provided a reliable evaluation of the model’s consistency through cross-validation and its real-world accuracy on unseen data. Each trained model was evaluated on four distinct datasets (Sentence, Counting, Days, and Combined), each reflecting different speech tasks. The comparative performance results for these datasets are presented in Table 9.

**Table 9:** Evaluation of voice model classification performance

Model	Sub-Dataset	Accuracy	Precision	Recall	$F_1$-Score	ROC-AUC
RF	Sentence	0.9557	0.9606	0.9557	0.9556	0.9906
	Counting	0.9640	0.9683	0.9640	0.9638	0.9968
	Days	0.9285	0.9340	0.9285	0.9283	0.9862
	Combined	0.9561	0.9620	0.9561	0.9558	**0.9985**
K-Nearest Neighbors	Sentence	0.9107	0.9211	0.9107	0.9101	0.9574
	Counting	**0.9731**	0.9754	**0.9731**	**0.9731**	0.9817
	Days	0.9379	0.9465	0.9379	0.9376	0.9886
	Combined	0.9727	**0.9763**	0.9726	0.9726	0.9934
Logistic Regression	Sentence	0.9194	0.9282	0.9194	0.9189	0.9116
	Counting	0.9466	0.9496	0.9466	0.9466	0.9412
	Days	0.9198	0.9334	0.9198	0.9189	0.9303
	Combined	0.9466	0.9481	0.9466	0.9466	0.9317

The bold values indicate the best-performing results for the corresponding evaluation metric

As shown in Table 9, model performance varies across datasets, with KNN achieving the highest accuracy (97.31%) on the “Counting” dataset. This model also reported strong scores across other metrics, including precision, recall, and F1-score. On the other hand, RF delivered the highest ROC-AUC value (0.9985) on the “Combined” dataset, indicating superior discriminative ability despite slightly lower accuracy (95.61%) compared to KNN. Logistic Regression yielded consistent yet lower performance across all datasets. Notably, all three models achieved their best or near-best accuracy values on the “Combined” dataset, highlighting the advantage of integrating multiple speech tasks. This suggests that combining distinct vocal inputs enhances the richness of the feature space and supports more robust model learning. These findings emphasize the significance of both model choice and dataset composition in optimizing voice-based classification systems. Based on these findings, the RF model trained on the “Combined” dataset was chosen for deployment due to its strong balance of accuracy and discriminative power.

**Fig. 10:**
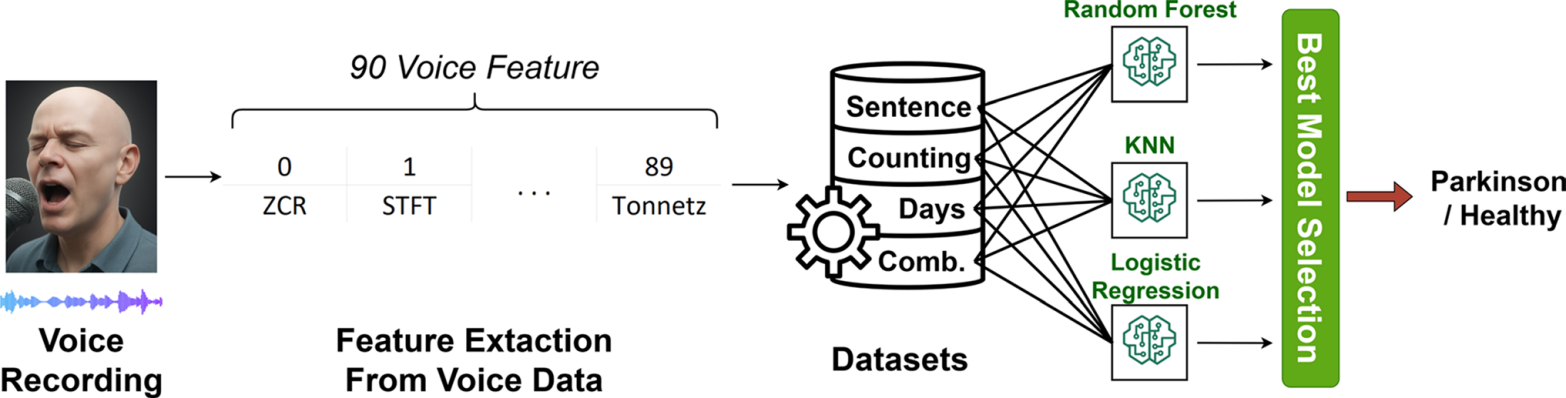
Voice modality model architecture

Consequently, the model could effectively learn both statistical and dynamic variations in the audio data. This choice was guided not only by accuracy but also by a systematic evaluation of all model-dataset combinations. Each dataset was trained with three ML algorithms, producing 12 distinct modeling scenarios. These were compared in terms of accuracy to identify the best-performing configuration and to understand which types of speech content worked best with specific models. This holistic approach aimed to ensure the model’s reliability and generalizability despite the limited data. The decision-making process and final classifier architecture are illustrated in Fig. 10.

### Posture modality module

We evaluated the posture modality module through two main processes: keypoint extraction unit and modeling, as detailed below.

#### Keypoint extraction

The module was developed with a focus on detecting Pisa Syndrome, one of the most common postural abnormalities observed in PD. Pisa Syndrome is characterized by abnormal lateral or forward tilting of the waist, neck, and back. Symptoms include excessive curvature of the spine, a lowered back posture, and abnormal head inclination.

To analyze participants’ postures, video recordings were captured using a mobile phone. These recordings focused on key stance components, including frontal posture, sagittal posture, and single-leg balance. The posture-related keypoints required for Pisa Syndrome detection were extracted from video frames using the MediaPipe library, which provides a comprehensive set of body landmarks (see Fig. 5). These keypoints were then used for further analysis of posture alignment and abnormalities.

We utilized the left shoulder, right shoulder, left ear, right ear, and left hip keypoints from the set provided by the MediaPipe library. These keypoints were used to calculate features relevant for Pisa Syndrome detection. Specifically, Eq. 16 was employed for two-dimensional feature extraction, while Eqs. 17 and 18 were used for three-dimensional feature extraction. Based on these calculations, a total of nine features were derived: two-dimensional and three-dimensional body rotation angles (shoulder slope), shoulder height difference, two-dimensional and three-dimensional neck slope angles, two-dimensional and three-dimensional trunk slope angles, and two-dimensional and three-dimensional ear slope angles.

Let $P_1 = (P_{1x},\ P_{1y})$ and $P_2 = (P_{2x},\ P_{2y})$ be two points in the 2D plane. The 2D angle representing the orientation from $P_1$ to $P_2$ is computed using the ${\text {atan2}}$ function, which returns the angle (in radians) between the x-axis and the vector from $P_1$ to $P_2$. The formula is given by:

16 $$\begin{aligned} 2D\ angle = \frac{atan2(P_{2y}-P_{1y},P_{2x}-P_{1x})\times 180}{\pi } - 90 \end{aligned}$$

Let $\boldsymbol{v}_1$ and $\boldsymbol{v}_2$ be two vectors in three-dimensional space. The angle $\theta$ between them is computed using the dot product formulation in Eq. 17, and the result is converted from radians to degrees in Eq. 18:

17 $$\begin{aligned} \theta = \cos ^{-1} \left( \frac{\boldsymbol{v}_1 \cdot \boldsymbol{v}_2}{\Vert \boldsymbol{v}_1\Vert \cdot \Vert \boldsymbol{v}_2\Vert } \right) \end{aligned}$$

18 $$\begin{aligned} 3D\ angle = \theta \cdot \frac{180}{\pi } \end{aligned}$$

**Fig. 11:**
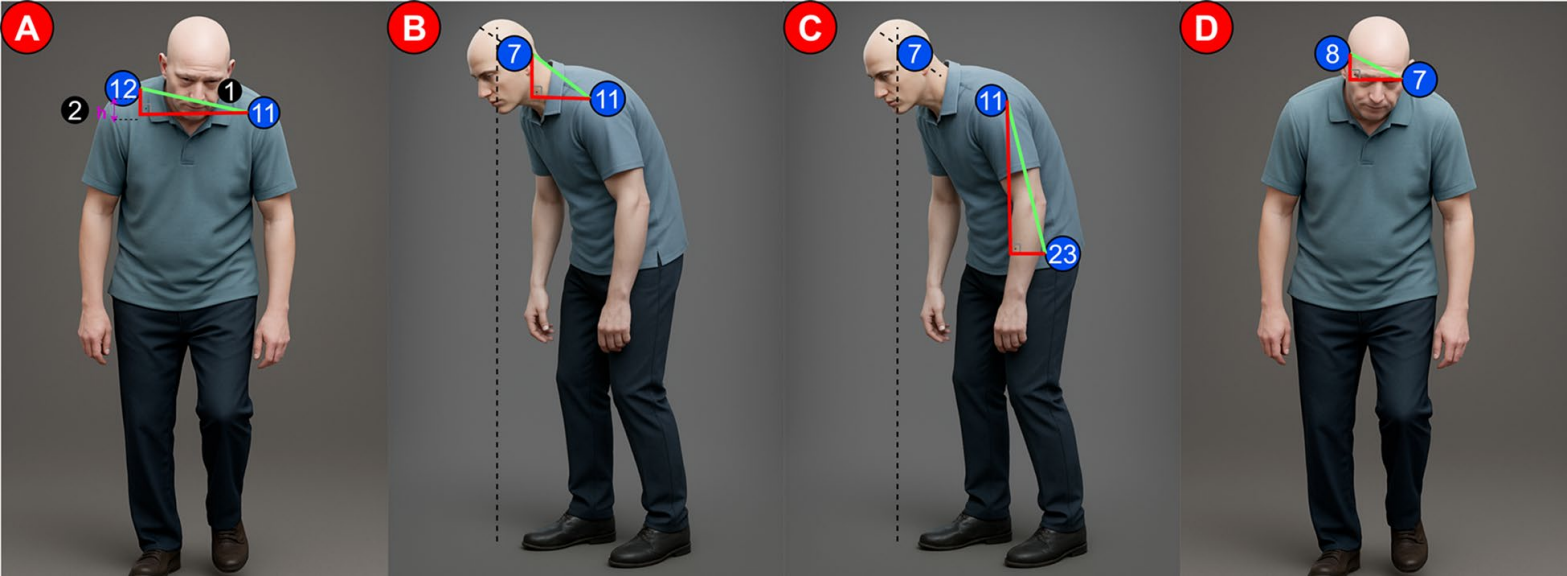
Postural analysis of pisa syndrome in the frontal and sagittal planes (**A.1**: The two-dimensional and three-dimensional body rotation angles, **A.2**: The difference in shoulder height, **B**: The two-dimensional and three-dimensional angles of neck slope, **C**: The two-dimensional and three-dimensional trunk slope angles, **D**: The two-dimensional and three-dimensional angles of the ear inclination

The two-dimensional and three-dimensional body rotation angles—also referred to as shoulder slope angles (Fig. 11.A.1)—represent the degree of shoulder tilt or rotation relative to the horizontal axis. These angles are calculated by measuring the 2D or 3D angle formed between the left and right shoulder keypoints. In a healthy posture, the shoulders are typically parallel to the ground and symmetrically aligned. However, any upward or downward deviation of one shoulder disrupts trunk alignment and postural symmetry. Such asymmetrical shoulder inclinations are characteristic indicators of postural abnormalities, including Pisa Syndrome.

The difference in shoulder height (Fig. 11. A.2) refers to the vertical disparity between the left and right shoulders. This feature is calculated by taking the absolute difference between the y-coordinates of the left and right shoulder keypoints. In a typical posture, both shoulders are level and aligned. However, when this symmetry is disrupted—such as when one shoulder is significantly higher or lower than the other—it often indicates pelvic misalignment or underlying musculoskeletal disorders. Shoulder height asymmetry not only compromises overall postural balance but can also lead to chronic waist and back pain over time.

The two-dimensional and three-dimensional angles of neck slope (Fig. 11.B) quantify the forward or backward tilt of the head relative to the shoulders. These angles are calculated using the 2D or 3D angle between the left ear and left shoulder keypoints. In a typical posture, the head aligns vertically with the shoulders. However, in patients with PD, excessive forward or backward neck inclination is common, disrupting head alignment and overall posture. Such deviations increase the load on neck and back muscles and contribute to balance issues and challenges in daily activities.

The two-dimensional and three-dimensional trunk slope angles (Fig. 11.C) measure the degree of forward or backward leaning of the trunk. These features are computed using the angle between the left hip and left shoulder keypoints in 2D and 3D space. Although healthy individuals generally maintain an upright trunk, Parkinson’s patients often exhibit characteristic inclinations toward or backward, indicating postural instability.

The two-dimensional and three-dimensional angles of the ear inclination (Fig. 11.D) represent the lateral tilt of the head. These angles are derived from the 2D or 3D angle between the left and right ear keypoints. Normally, both ears are horizontally aligned. However, in Parkinson’s patients, the head may tilt abnormally to one side, increasing the angle between the ears and indicating significant postural asymmetry.

To identify postural irregularities, each movement was assessed from three distinct video perspectives, summarized as follows:

- **Short:** Captures only the sagittal posture.
- **Long:** Includes both sagittal and frontal postures.
- **Full:** Covers the entire range of motion, including frontal posture, sagittal posture, and single-leg stance.

For each video type, we constructed three separate datasets containing 9 keypoint-based features per video frame. To extract meaningful information from these sequential data, we utilized the TSFEL (Time Series Feature Extraction Library). TSFEL computes a wide range of time series features, including statistical features that describe the overall structure of the motion, temporal features that reflect the distribution of motion over time, and frequency features that capture the periodic components of the movement. As a result, each dataset yielded 1404 features derived from the 9 frame-level measurements.

#### Modeling

**Fig. 12:**
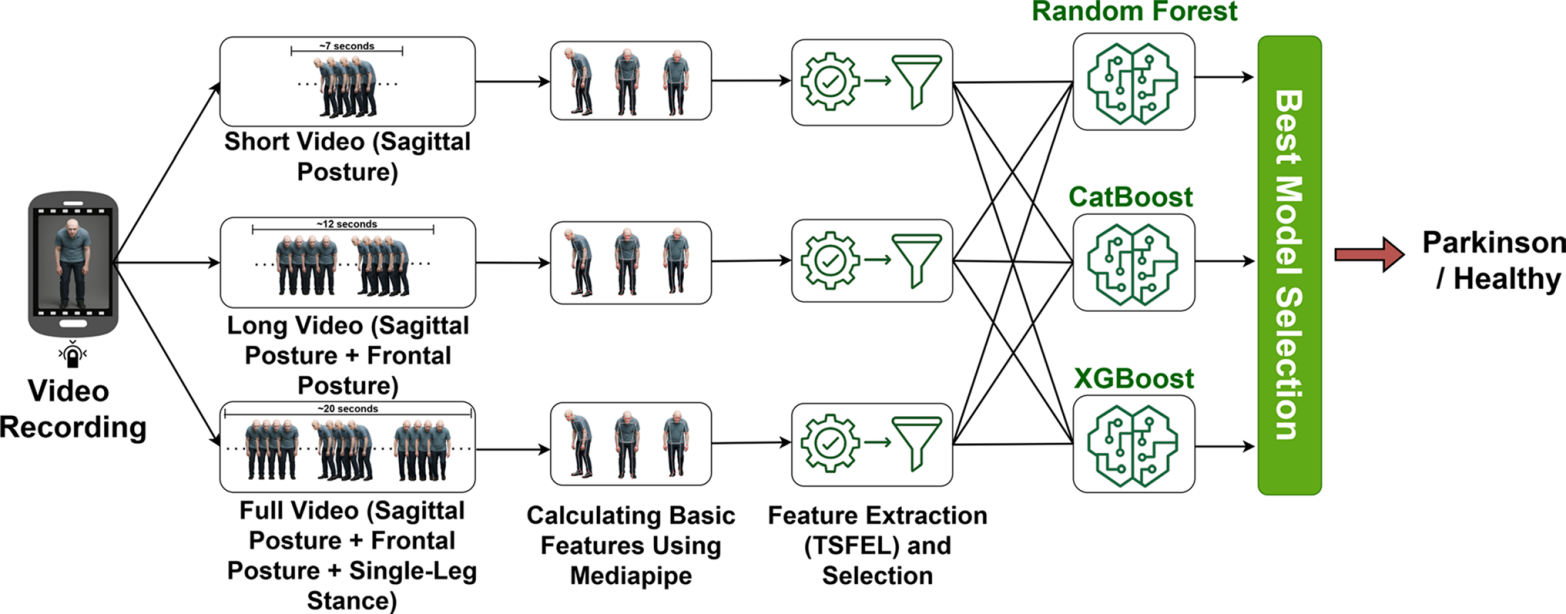
Posture modality model architecture

Three distinct datasets were created based on different video types. The dataset, derived from short videos, includes 20 healthy individuals and 37 patients with PD. Similarly, the dataset from long videos comprises 20 healthy individuals and 37 Parkinson’s patients. The dataset, based on full-length videos, contains data from 20 healthy individuals and 32 patients with PD. The lower number of Parkinson’s patients in the full video dataset is due to five patients being unable to perform the single-leg stance without support. The features extracted from the three datasets—each comprising 1,404 features—were evaluated using four different feature selection methods prior to being used as model inputs. For each dataset, features were ranked based on their importance using the following techniques: RF with Feature Importance, Logistic Regression with Lasso, SelectKBest, and RFECV (Recursive Feature Elimination with Cross-Validation). The top 30 features with the highest scores were selected for subsequent modeling. All datasets were split into 80% training and 20% test sets to maintain a balanced distribution of label values. To augment the data, Gaussian noise was added, effectively doubling the number of samples. Additionally, to address class imbalance between healthy individuals and Parkinson’s patients, the SMOTE method was applied to equalize the class distributions. During the modeling phase, three different pipeline structures were constructed. In each pipeline, the data were first standardized using StandardScaler, followed by classification using one of the following models: RF, CatBoost, or XGBoost. The architecture of the system for the posture modality is illustrated in Fig. 12.

To identify the best-performing model, a comprehensive hyperparameter optimization process was conducted. The most suitable parameter combinations for each model were systematically evaluated based on training accuracy and cross-validation performance. A three-fold cross-validation (K=3) was applied to the training data, and the hyperparameter sets that yielded the highest accuracy and best generalization ability were selected for the final models. The hyperparameter search space used in this process is presented in Table 10.

**Table 10:** Hyperparameter search space for posture module

Model	Hyperparameter	Possible Values
RF	n_estimators	[20, 30, 50]
	max_depth	[None, 3, 5, 7]
	min_samples_split	[5, 8]
	min_samples_leaf	[2, 4]
CatBoost	iterations	[30, 50]
	learning_rate	[0.005, 0.01]
	depth	[2, 3]
	l2_leaf_reg	[5, 7, 10]
XGBoost	n_estimators	[30, 50]
	learning_rate	[0.005, 0.01]
	max_depth	[2, 3]
	reg_lambda	[1, 3, 5, 7]

The top 30 highest-scoring features extracted from the “Short”, “Long”, and “Full” video types were input into the constructed pipelines, and the performance of three different models was compared. The results of this comparison are presented in Table 11. According to the table, the highest classification performance is obtained from the RF posture model trained on full-length video recordings. This result can be attributed to the inclusion of frontal posture, sagittal posture, and single-leg stance within the same recording, which allows the model to capture a wider range of posture-related abnormalities associated with PD.

**Table 11:** Evaluation of posture model classification performance

Model	Sub-Dataset	Accuracy	Precision	Recall	$F_1$-Score	ROC-AUC
RF	Short	0.9318	0.9018	0.9000	0.8999	0.9555
	Long	0.9321	0.8947	0.8667	0.8643	0.9467
	Full	**0.9464**	0.8949	0.8929	0.8927	**0.9745**
CatBoost	Short	0.8474	0.9018	0.9000	0.8999	0.9289
	Long	0.8637	0.8750	0.8333	0.8286	0.9079
	Full	0.9106	0.8889	0.8571	0.8542	0.9412
XGBoost	Short	0.8729	0.9018	0.9000	0.8999	0.9023
	Long	0.9235	0.8733	0.8667	0.8661	0.9461
	Full	0.8483	0.7917	0.7857	0.7846	0.9292

The bold values indicate the best-performing results for the corresponding evaluation metric

### Comparative summary of experimental results

In this subsection, we present a detailed summary of our experimental outcomes and conduct a comparative evaluation with previously published related studies.

Table 12 offers a comprehensive overview of previous work on Parkinson’s disease detection, detailing the datasets used, the best-performing models proposed in each study, and their associated performance metrics. It also compares these existing approaches with the performance of our proposed models across different data modalities. Note that, since no model was trained in [2] and [10], and a holistic evaluation was conducted in [9], these studies were not included in the table. In addition, performance metrics that are not provided in the studies are indicated with a "-" symbol.

According to the table, in the walking modality, our Bidirectional GRU model achieved superior results compared to previous studies, with 92.68% accuracy, 93.39% precision, 92.38% recall, 92.74% $F_1$-score, and an AUC of 97.86%. This model notably outperformed other approaches. For face modality, our ensemble model—combining XGBoost, RF, and CatBoost—demonstrated strong performance with 92.86% accuracy and perfect precision (100%), as well as balanced metrics including 85.71% recall, 92.31% $F_1$-score, and 97.96% AUC. Although the EfficientNet-B7 model given in [7] reported a perfect accuracy of 100%, the absence of other key evaluation metrics calls its generalizability into question. In the voice modality, our RF-based model was particularly noteworthy, achieving 95.61% accuracy and the highest AUC value among all modalities at 99.85%, along with a balanced $F_1$-score of 95.58%. Although all performance metrics for the SVM model given in [5], except for AUC, were reported as 100%, the study did not provide the AUC value. As a result, the model’s ability to distinguish between classes cannot be clearly assessed. For posture-based classification, our RF model recorded 94.64% accuracy and a 97.56% AUC. While its accuracy was slightly lower than the KNN model reported in [3], its overall performance remains highly competitive. Overall, our proposed models demonstrate consistent improvements across all modalities, with high accuracy, balanced precision-recall metrics, and strong AUC scores. These findings affirm that our methods offer reliable and effective classification of PD across various modalities.

**Table 12:** Model performance across modalities

Modality	Reference	Dataset	Model	Accuracy	Precision	Recall	$F_1$score	AUC
Walking	[12]	102 PD 46 HC	CST-GCN + FST-GCN	0.677	0.666	0.641	0.647	0.803
	[15]	43 PD 37 HC	(SSF-CNN)	0.7125	0.713	0.710	0.710	0.789
	[17]	30 PD 42 HC	ResNet50_3.4 + RF	0.9167	-	-	-	-
	Ours	21 PD 20 HC	Bidirectional GRU	**0.9268**	**0.9339**	**0.9238**	**0.9274**	**0.9786**
Face	[7]	95 PD 380 HC	EfficientNet-B7	**1.0**	-	-	-	-
	[8]	186 PD 185 HC	RF	-	-	-	-	0.69
	[14]	51 PD 55 HC	ResNet34 + LSTM	0.8173	0.9684	0.7224	0.8275	-
	Ours	38 PD 21 HC	XGBoost + RF + CatBoost	0.9286	**1.0**	**0.8571**	**0.9231**	**0.9796**
Voice	[4]	16 PD 21 HC	WNN	0.923	0.92	0.92	0.92	-
	[5]	30 PD 25 HC	SVM	**1.0**	**1.0**	**1.0**	**1.0**	-
	[8]	117 PD 50 HC	AdaBoost	-	-	-	-	0.86
	[11]	47 PD 48 HC	SVM – linear kernel	0.983	0.966	1.0	-	-
	Ours	35 PD 21 HC	RF	0.9561	0.9620	0.9561	0.9558	**0.9985**
Posture	[16]	70 PD	Optimal Desicion Tree	0.9000	0.8910	-	-	-
	[3]	20 PD 15 HC	KNN	**0.956**	**0.952**	**0.931**	**0.942**	0.95
	Ours	35 PD 19 HC	RF	0.9464	0.8949	0.8929	0.8927	**0.9756**

The bold values indicate the best-performing results for the corresponding evaluation metric

Taken together, the results in Table 12 highlight that most existing studies address PD detection in a modality-specific manner, whereas the proposed framework consistently demonstrates strong performance across all evaluated modalities.

## System integration

This section details the integration process of the AI-assisted decision support system developed for the early diagnosis of PD. The system architecture enables the seamless fusion of analytical outputs from four distinct modalities—walking, facial expression, voice, and posture—to generate a final diagnostic decision. To facilitate user interaction, a Gradio-based web application was developed, providing a user-friendly interface for accessing model predictions. The integration process was designed with a modular structure to optimize outputs from diverse model architectures, improve interpretability, and support real-time applications.

**Fig. 13:**
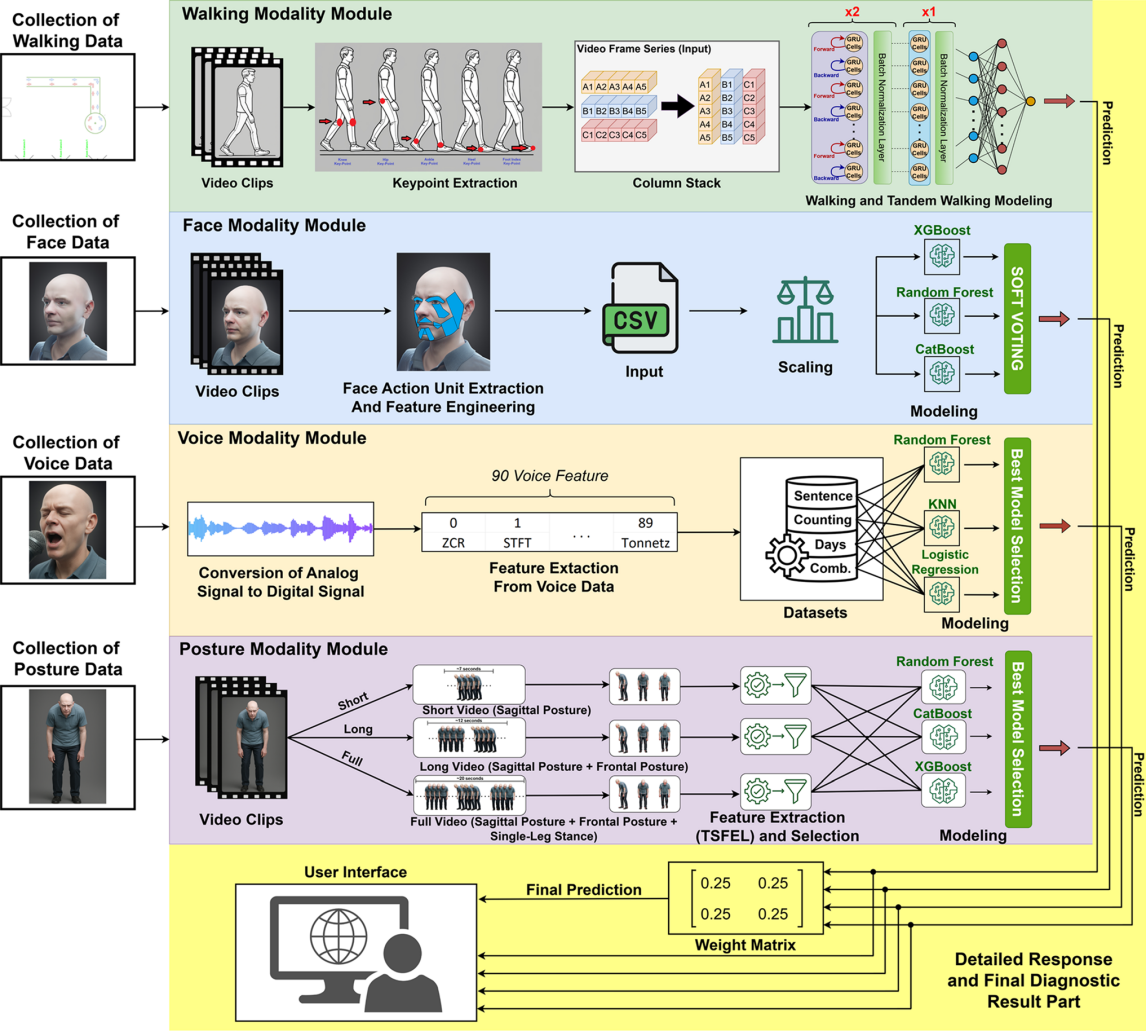
Overview of the integrated AI-assisted decision support system for early PD diagnosis. The architecture combines outputs from four modality-specific modules—walking, facial expression, voice, and posture—and fuses them through a modular pipeline to produce the final diagnostic decision

The complete system architecture is illustrated in Fig. 13. According to the figure, the final decision-making process of the modular diagnostic support system is based on the weighted average of prediction probabilities obtained from four independent classifiers: walking, face, voice, and posture. The inter-modality weighting was determined in consultation with a specialist from the Department of Neurology at Kocaeli University, ensuring that medical expertise was incorporated into the system’s decision-making logic. Based on this clinical guidance, equal weights of 25% were assigned to each module. Each modality-specific prediction score was multiplied by its corresponding weight, and the weighted scores were summed to compute a final decision score. This score was then compared against a threshold value of 0.5. If the score exceeded the threshold, the individual was classified as having PD; otherwise, they were classified as healthy. The threshold was selected in accordance with common binary classification practices and optimized for a balanced trade-off between sensitivity and specificity. This approach enables the comprehensive integration of multimodal data, mitigating uncertainties that may arise from single-modality outputs and enhancing the system’s generalization capability. Furthermore, the decision-making framework is transparent, clinically interpretable, and aligned with the principles of explainable AI. Rather than being a mere mathematical aggregation, the final decision represents an informed synthesis of diverse model outputs shaped by expert knowledge. As such, the system delivers reliable, medically grounded diagnostic support that is suitable for both user-facing interfaces and clinical use.

**Fig. 14:**
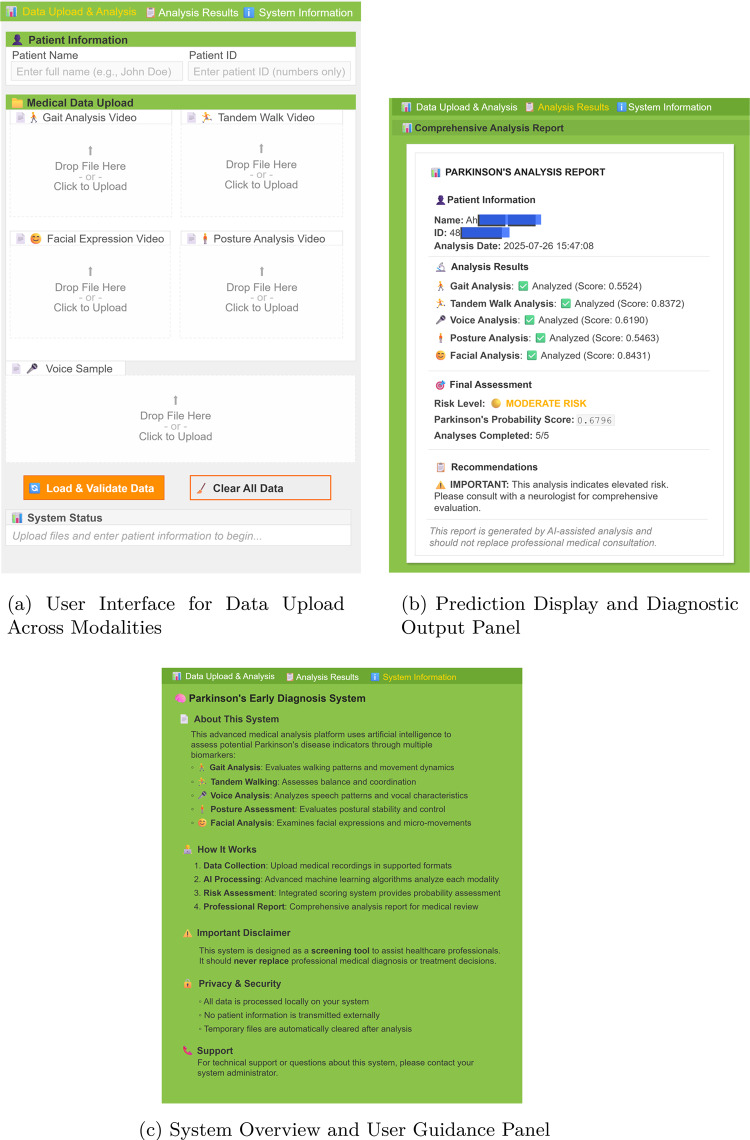
Graphical user interface tabs

The developed diagnostic support system has been integrated with a Gradio-based web interface to enhance user interaction and make the decision-making process more visual and accessible. This interface is designed to streamline usage for both clinicians and researchers. Each modality is represented by a dedicated input section, enabling users to sequentially upload walking video, facial video, voice recording, and posture video. After data upload is completed, the system computes individual prediction scores for each modality and displays these scores numerically. In parallel, the system integrates the scores using the underlying weighted decision model, calculates the final decision score, and presents the diagnostic result as either “Parkinson” or “Healthy.” Additionally, the interface incorporates a feedback mechanism that allows clinicians to enter their final diagnosis and submit it to the system for continuous evaluation and improvement. The structure and functionality of the interface are illustrated in Figs. 14a and b. Figure 14c provides a comprehensive overview of the system’s functionality, detailing each analysis modality—including walking, tandem walking, voice, posture, and facial analysis. The workflow section clearly describes the process: users upload their data, the AI model analyzes it, and the system generates a risk assessment accompanied by a professional report. A disclaimer emphasizes that the tool is intended for screening purposes only and is not a substitute for medical diagnosis. Furthermore, it includes privacy and security information, highlighting that all data is processed locally and that temporary files are deleted automatically. Contact details are also provided to support users in case of questions or technical issues.

## Conclusion

This study introduces an innovative AI-assisted decision support system designed for the early diagnosis of idiopathic PD. Leveraging multimodal data sources—including walking recordings, facial expressions, voice recordings, and posture—the system combines deep learning, ML, and ensemble learning techniques to deliver a robust and reliable diagnostic framework. The proposed system demonstrates high classification performance and exhibits strong potential for clinical adoption.

A key contribution of this work is the synchronized integration of four heterogeneous data modalities within a unified diagnostic framework. To the best of our knowledge, this is the first study to incorporate all four modalities simultaneously, effectively addressing the limitations of conventional unimodal or bimodal diagnostic approaches. By leveraging this diverse set of clinical assessments, the system substantially improves diagnostic accuracy while preserving interpretability for clinical users.

Although the results are promising, the study has several limitations, among which the need for larger and more diverse datasets emerges as a critical factor to improve the model’s generalizability. Furthermore, real-time deployment and adaptation to varying clinical settings warrant further investigation. As part of future research, we aim to improve the system’s scalability, optimize its computational efficiency, and expand the dataset to encompass more diverse populations.

Despite the promising results of this study, several limitations should be acknowledged. First, the dataset was collected from a single clinical center and included a relatively limited number of participants, which may affect the generalizability of the findings. In addition, although the proposed system demonstrates strong performance in an offline evaluation setting, its real-time deployment and robustness across diverse clinical environments remain to be investigated. Future work will focus on expanding the dataset through multicenter collaborations, improving model generalization across heterogeneous populations, and optimizing the framework for real-time clinical use, with the ultimate goal of enabling large-scale and reliable early screening of PD.

## Supplementary Information

Below is the link to the electronic supplementary material.

## Data Availability

The source code utilized in our experiments are available via the following link: https://github.com/Bakwein/Early-Diagnosis-of-Idiopathic-Parkinsons-Disease-Using-an-Artificial-Intelligence-Assisted-System.
